# Controversial Role of Transferrin in the Transport of Ruthenium Anticancer Drugs

**DOI:** 10.3390/biom12091319

**Published:** 2022-09-18

**Authors:** Aviva Levina, Anthony R. M. Chetcuti, Peter A. Lay

**Affiliations:** 1 School of Chemistry, The University of Sydney, Sydney, NSW 2006, Australia; 2 School of Sydney Analytical, The University of Sydney, Sydney, NSW 2006, Australia

**Keywords:** ruthenium, KP1019, anticancer, transferrin, albumin, transferrin receptor, biolayer interferometry, HepG2 cells, protein aggregation, intratumoral injections

## Abstract

Ruthenium complexes are at the forefront of developments in metal-based anticancer drugs, but many questions remain open regarding their reactivity in biological media, including the role of transferrin (Tf) in their transport and cellular uptake. A well-known anticancer drug, KP1019 ((IndH)[Ru^III^Cl_4_(Ind)_2_], where Ind = indazole) and a reference complex, [Ru^III^(nta)_2_]^3−^ (nta = nitrilotriacetato(3−)) interacted differently with human apoTf, monoFeTf, or Fe_2_Tf. These reactions were studied by biolayer interferometry (BLI) measurements of Ru–Fe–Tf binding to recombinant human transferrin receptor 1 (TfR1) in conjunction with UV-vis spectroscopy and particle size analysis. Cellular Ru uptake in human hepatoma (HepG2) cells was measured under the conditions of the BLI assays. The mode of Tf binding and cellular Ru uptake were critically dependent on the nature of Ru complex, availability of Fe(III) binding sites of Tf, and the presence of proteins that competed for metal binding, particularly serum albumin. Cellular uptake of KP1019 was not Tf-mediated and occurred mostly by passive diffusion, which may also be suitable for treatments of inoperable cancers by intratumoral injections. High cellular Ru uptake from a combination of [Ru^III^(nta)_2_]^3−^ and Fe_2_Tf in the absence of significant Ru–Tf binding was likely to be due to trapping of Ru(III) species into the endosome during TfR1-mediated endocytosis of Fe_2_Tf.

## 1. Introduction

The resounding clinical success of Pt-based anticancer drugs (cisplatin, carboplatin, and oxaliplatin) led to considerable interest in the use of transition metal complexes for cancer chemotherapy, but no comparable breakthroughs for other metal complexes have occurred in the field so far [1,2]. Ruthenium compounds are currently at the forefront of preclinical development in metal-based anticancer drugs [1,2,3,4,5,6]. Unique photophysical properties of Ru can lead to its applications in light-activated anticancer drugs and in photodynamic therapy [7]. Some Ru complexes showed immunogenic properties that are particularly promising for cancer immunotherapy [8,9].

One of the main problems for medicinal applications of Ru complexes and metal-based drugs in general is their complicated and unpredictable reactivity in biological media both extra- and intra-cellularly [10,11,12,13,14]. For instance, NAMI-A ((ImH)[Ru^III^Cl_4_(Im)(*S*-dmso)], where Im = imidazole and *S*-dmso is *S*-bound dimethyl sulfoxide) showed great promise as a selective antimetastatic drug in preclinical studies but failed in phase I/II human clinical trials [15], probably due to non-specific binding to extracellular targets [16]. A related Ru(III) complex, KP1019 ((IndH)[Ru^III^Cl_4_(Ind)_2_], where Ind = indazole) was withdrawn from early clinical trials due to insufficient solubility in biological media [1]. However, its more soluble analogue with a Na^+^ counterion (known as NKP-1339, IT-139 or BOLD-100) remains in clinical trials (NCT01415297 and NCT04421820) [17] and shows promising activities, including immunogenic effects and alteration of glycolysis in cancer cells [1,18,19,20].

One of the outstanding questions in the development of Ru anticancer drugs (RuACDs) is the role of transferrin (Tf, the main Fe transport protein in mammalian blood) [21] in cellular Ru uptake. Transferrin is an ~80 kDa glycoprotein that consists of two structurally similar lobes (*C*- and *N*-lobes), each of which binds one Fe(III) ion in a pocket that consists of two tyrosine, one aspartate, one histidine residues and a synergistic carbonato anion [21]. Initial studies suggested that selective binding of Ru(III) to the Fe(III) binding sites of Tf may be responsible for efficient cellular uptake and anticancer activity of Ru(III) complexes, particularly for KP1019 [22,23]. More recently, Ru(III) binding to the Fe(III) binding sites of Tf was shown to disrupt the binding of Tf to its main cellular receptor (transferrin receptor 1, TfR1) [24]. Any cellular uptake of Ru–Tf adducts is more likely due to non-specific Ru binding to the side chains of fully Fe(III)-saturated Tf (holoTf, Fe_2_Tf) [25,26].

Our group has pioneered the use of biolayer interferometry (BLI) [24,27] in combination with previously developed urea gel electrophoresis [28,29] and other analytical techniques for elucidation of the roles of non-Fe metal binding to Tf in their cellular uptake and biological activity. Previously, this approach was used for the studies of Tf interactions with NAMI-A [24], as well as with Cr(III) [30] and V(V/IV) [31] complexes. This work presents a detailed study of Tf interactions with a well-known anticancer Ru(III) complex, KP1019, which has been the subject of contradictory claims in the literature about its potential role in anticancer activities in vivo [23,32]. Herein, we studied the role of proteins that compete for Ru binding in vitro and in vivo, in particular serum albumin, in modulating Ru–Tf binding and cellular Ru uptake under physiologically relevant conditions.

## 2. Materials and Methods

### 2.1. Reagents and Solutions

The following sources of proteins and reagents were used: (i) recombinant human transferrin receptor (TfR1/CD71), extracellular domain (Cyst89-Phe760), (His)_6_-tagged at the *N*-terminus, from Sino Biological, Beijing, China (Cat No.11020-H07H); (ii) human apo transferrin (apoTf) from Sigma-Aldrich, Burlington, MA, USA (>98% Tf ≤ 0.005% Fe; Cat No T1147); (iii) bovine serum albumin (BSA) with high fatty acid content (AlbuMax, Gibco Cat No 11020021) for cell assays; (iv) human serum albumin (HSA, >99%, Sigma-Aldrich A3782)); (v) human serum (sterile filtered) Sigma Cat. No. H4522; and (vi) 1000 ppm standard RuCl_3_ solution in 5% HCl and 1000 ppm standard FeCl_3_ in 0.1 M HCl solution (>99%, Sigma-Aldrich). Representative anticancer Ru complexes, NAMI-A, KP1019, and RAPTA-C ([Ru^II^(η^6^-*p*-cymene)Cl_2_(pta)], where pta = 1,3,5-triaza-7-phosphoadamantane), were synthesized according to the literature methods [33,34,35] and characterized by nuclear magnetic resonance spectroscopy (^1^H NMR), electrospray ionization mass spectrometry (ESI-MS), and elemental analysis, as reported previously by our group [36,37]. Stock solutions of Ru complexes (10–20 mM) in *N*,*N*-dimethyl formamide (DMF) [36] were prepared on the day of experiments. The use of dimethyl sulfoxide (DMSO) for stock solutions was avoided because of its known ligand-exchange reactions with Ru(III/II) complexes [38]. All other reagents were of the highest available purity grade and purchased from Sigma-Aldrich or Merck. Sterile solutions and plasticware used in cell culture were purchased from Life Technologies Australia. Milli-Q water was used in all aqueous sample preparations.

The buffers used for metal–Tf binding studies [24,27,28,29,30,31] were as follows: (i) the binding buffer (20 mM HEPES, 25 mM NaHCO_3_, 140 mM NaCl, pH 7.4; HEPES = 4-(2-hydroxy ethyl)piperazine-1-ethanesulfonic acid); and (ii) the endosomal buffer (100 mM MES, 300 mM KCl, 4.0 mM Na_2_H_2_edta, pH 5.6; MES = 2-(*N-*morpholino)ethanesulfonic acid, edta = *N*,*N*,*N′*,*N′*-ethane-1,2-diaminetetraacetate(4−)). To minimize the effect of trace Fe(III) on metal–Tf binding, the buffers were pre-treated with Chelex 100 chelating resin (BioRad, Contra Costa County, CA, USA) for 36 h, whilst adjusting the pH values with solutions of ultra-pure HCl (0.10 M, Merck, Kenilworth, NJ, USA), or NaOH (0.10 M, Aldrich, St. Louis, MO, USA) then filtered through sterile 0.2 µM membrane filters (Sartorius) before use. The pH values of the buffers were measured with an Activon 210 pH meter equipped with a combined glass/Ag/AgCl electrode (model No H11332) and calibrated using standard pH buffer solutions.

### 2.2. Metal–Tf Sample Preparation

Conditions used for Fe(III)–Tf and Ru(III)–Tf sample preparations are listed in Table 1. Aqueous solutions of the precursor [Fe^III^(nta)_2_]^3−^ and [Ru^III^(nta)_2_]^3−^ complexes (~10 mM metal, pH~6 for Fe(III) or pH~2 for Ru(III), nta = nitrilotriacetato(3−)) were prepared according to the literature methods [39,40]. Precise Fe(III) or Ru(III) concentrations in these solutions were determined by graphite furnace atomic absorption spectrometry (GFAAS), using an Agilent Technologies series 200 spectrometer equipped with Zeeman background correction. Unless stated otherwise, metal–Tf solutions for electronic absorption (UV-vis) spectroscopy and BLI studies (A1–A9 in Table 1) were prepared in the binding buffer (Section 2.1) and incubated for 24 h at 295 K before the experiments. The UV-vis spectra of Tf solutions (~0.1 mM protein) were collected using a Thermo Fisher Scientific NanoDrop spectrometer (200–750 nm spectral range, ~3 nm wavelength resolution, 2.0 μL sample size). The following literature values were used to determine Tf concentrations: *λ*_max_ = 280 nm, *ε*_max_ = 8.5 × 10^4^ M^−1^ cm^−1^ for apoTf; *λ*_max_ = 280 nm, *ε*_max_ = 1.04 × 10^5^ M^−1^ cm^−1^ for Fe_2_Tf and Ru_2_Tf; *λ*_max_ = 470 nm, *ε*_max_ = 4.9 × 10^3^ M^−1^ cm^−1^ for Fe_2_Tf; and *λ*_max_ = 380 nm, *ε*_max_ = 7.0 × 10^3^ M^−1^ cm^−1^ for Ru_2_Tf [39,40,41].

Freeze-dried samples of Fe_2_Tf (fully Fe-saturated) or Fe_0.6_Tf (30% saturated, which corresponded to the mean Fe–Tf saturation in human blood) [42] for cell culture assays (B1–B9 in Table 2) were prepared by the reactions of apoTf with Fe(III)–NTA. These metalloproteins were characterized by UV-Vis spectroscopy and Fe content measurements by GFAAS, as described previously [30]. Similarly, a sample of Ru_2_Tf was prepared by mixing a solution of human apoTf (10 mg in 0.50 mL of the binding buffer) with that of [Ru^III^(nta)_2_]^3−^ (0.15 mL of 2.02 mM solution in water, a 5-fold molar excess Ru). The mixture was incubated for 2 h at 310 K, followed by removal of low-molecular-mass Ru species (<3 kDa) using Nanosep centrifugal membrane filters (Pall Life Sciences Cat. No. OD003C34, New York, NY, USA) and the resultant solution was freeze-dried [30].

### 2.3. Biolayer Interferometry Measurements

The effects of Ru complexes on the Tf cycle were determined by BLI using previously developed protocols [24,27,30,31]. The instrument used was a single-channel BLItz analyser (ForteBio, Menlo Park, CA, USA) with a tube holder (250 µL solution volume) and Ni(II)–nta coated optical probes held at 295 ± 1 K. Measurements used the Advanced Kinetics mode. The probes were loaded with TfR1 (250 mg mL^−1^) in phosphate buffered saline solution (PBS) and were re-used up to forty times after a single loading, which did not cause significant changes in the kinetics of Tf binding [24]. Solutions of metal–Tf adducts (typical final Tf concentrations 150 nM or 1.0 µM) were prepared in the binding buffer (Section 2.1). The pH 7.4 binding buffer was used for Tf binding and dissociation from the probe, and the pH 5.6 buffer (Section 2.1) was used to mimic the endosomal step of the Tf cycle [27]. Metal–Tf solutions (A1–A9 in Table 1) were diluted 100–400-fold, cell culture media were diluted 30-fold, and human serum samples were diluted 200-fold with the binding buffer immediately before the BLI measurements. All BLI data were background corrected by using data from buffer only runs with the same TfR1-loaded optical probe. Consistent BLI results were obtained in at least two independent measurements, using different probes and different batches of Tf solutions. Calculations of the Tf–TfR1 binding constants were performed using the 1:1 binding model (BLItz software, Version 1.1, Forte-Bio 2013) [24,30], which can be used as a reasonable approximation of the physiological 2:2 Tf–TfR1 binding mechanism at low Tf concentrations [27,43,44,45].

### 2.4. Protein Aggregation Measurements, Gel Electrophoresis, and Crystal Structure Analysis

Changes in size of proteins following the reactions of apoTf and Fe_2_Tf with Ru complexes were determined by a dynamic light scattering (DLS) technique, using a Malvern ZetaSizer NanoS instrument (173° scattering angle, 298 K) with ZEN0040 disposable cuvettes (Malvern Panalytical, Malvern, UK). Protein samples (0.10 mM) were diluted 10-fold with binding buffer before the measurements. The measured parameters were the averages of 12–15 scans (scan time, 3 s). Urea gel electrophoresis of apoTf and Fe_2_Tf samples [28] in the presence of varying concentrations of KP1019 was performed as described previously [29]. Analysis of Tf crystal structures that are available in the public domain from the Protein Data Bank (PDB) was performed with PyMOL software (version 2.1.1, Schrodinger LLC 2021, New York, NY, USA).

### 2.5. Cell Culture and Treatment

Human hepatoma (HepG2) cells were originally received from the American Type Culture Collection (ATCC, Cat. No. HB-8065) and cultured as described previously [30]. This cell line was used for Tf-dependent metal uptake studies [30] because of the high expression levels of human TfR1 [43]. For treatments, the cells were grown to confluence in 24-well plates, using Advanced DMEM (Dulbecco’s modified Eagle’s minimal essential medium, Invitrogen Cat. No. 12491-015), supplemented with L-glutamine (2.0 mM), antibiotic-antimycotic mixture (100 mg mL^−1^ penicillin, 100 mg mL^−1^ steptomycin and 0.25 mg mL^−1^ amphotericin B) and fetal calf serum (heat-inactivated, 2% *v*/*v*). Confluent cell layers were washed with serum-free treatment medium (DMEM, Invitrogen Cat. No. 19938-025), supplemented with glutamine, antibiotic-antimycotic mixture and 1.0 mg mL^−1^ AlbuMax (Gibco Cat. No. 11020-021). Advanced DMEM was not used for the treatments, because it contained added proteins and growth factors, including low concentrations of human Fe_2_Tf [11]. After washing, the wells were filled with the treatment medium containing metal additions (detailed in B1–B9 of Table 1). The treatment media were sterilized by filtration through 0.2 µm membrane filters, and pre-equilibrated for 24 h at 310 K and 5% CO_2_ prior to the addition to cells, except for sample B1 that was used immediately after preparation. All the treatments were performed in triplicate, using random well positions to avoid experimental bias.

### 2.6. Measurements of Cellular Ru and Fe Uptake

After 24 h incubation with the treatment media (B1–B9 in Table 1), the medium from each well was collected for the determination of Ru–protein binding and BLI analyses, and the wells were washed twice with PBS (0.50 mL/well). The cells were then lysed with NaOH (0.10 M, 0.10 mL per well) for 3 d at 277 K. A 25 µL aliquot of each cell lysate was mixed with HCl (975 µL, 0.10 M) and kept for 24 h at 295 K, followed by centrifugation for 5 min at 16,000 g. The supernatants were used for Ru and Fe determination by ICP-MS using a Perkin-Elmer Nexion 350X spectrometer with standard Ru and Fe solutions (1.0–100 ppb) and ^193^Ir peak as an internal standard.

The values of metal uptake were normalized using protein content in cell lysates. For protein determination, Bradford reagent (Sigma Cat. No B6916; 98 µL) was mixed with a small aliquot (2.0 µL) of each cell lysate, and the absorbance at 600 nm was measured using a Victor V3 plate reader. The instrument was calibrated using solutions prepared by mixing BSA (2.0 µL, 0–2 mg mL^−1^ in 0.10 M NaOH, freshly prepared) with Bradford reagent (98 µL). The test and calibration samples were analyzed in triplicate. Statistical analysis of results was performed using one way ANOVA in Origin software (version 6.1, MicroCal, Northampton, MA, USA, 1999).

### 2.7. Determination of Ru–Protein Binding in Cell Culture Medium

An aliquot (50 µL) of cell culture medium from each well was passed through a separate Bio-Rad P6 centrifugal gel filtration column that was pre-saturated with PBS, according to manufacturer’s instructions. The eluants (50 ± 5 µL volume) contained protein-bound Ru fractions (>6 kDa molecular mass) [30,31]. The protein recovery, measured by Bradford assays, exceeded 95%. Another 50 µL sample of each medium was taken into separate tubes. Both the original and gel-filtered media samples were digested with 0.20 mL of 65% (*v*/*v*) HNO_3_ for 24 h. The digests were diluted with 0.75 mL of 0.10 M HCl, and the Ru content in the samples was determined by GFAAS. The percentage of protein binding was determined from the ratio of Ru content in the gel-filtered and original media samples [30,31].

## 3. Results

### 3.1. Effect of Ru Complexes on Tf–TfR1 Binding in Cell-Free Systems

**Figure 1:**
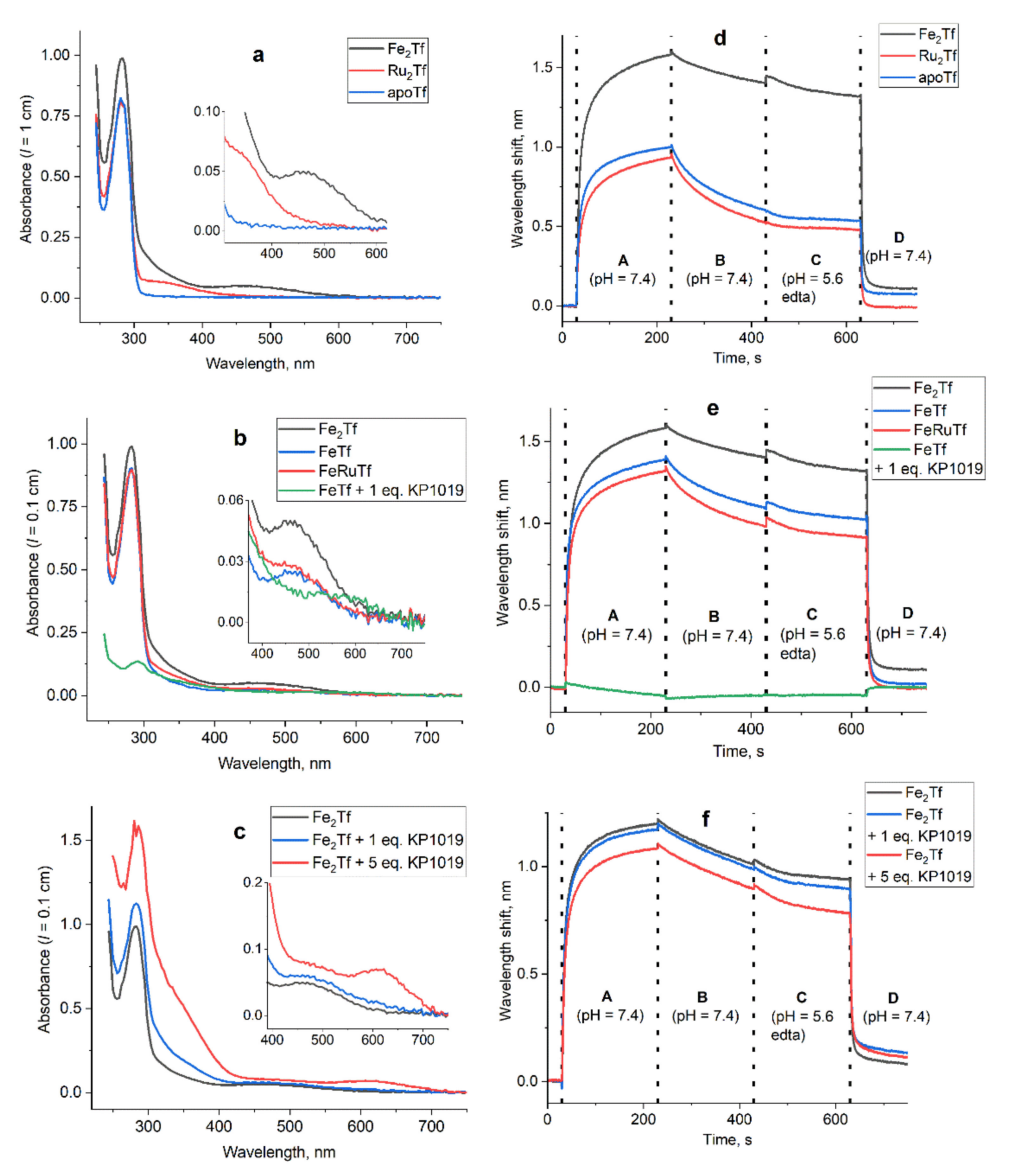
Representative UV-vis spectra (**a**–**c**) and the corresponding BLI curves (**d**–**f**) of Fe(III)–Ru(III)–Tf samples. Designations of the samples correspond to those used in Table 2. Tf concentrations were 0.10 mM for UV-vis spectra and 1.0 μM for BLI experiments. The buffer used in UV-vis spectra and in the steps A, B and D of BLI experiments was 20 mM HEPES, 25 mM NaHCO_3_, 140 mM NaCl, pH 7.4. The buffer used in step C of BLI experiments was 100 mM MES, 300 mM NaCl, 4.0 mM Na_2_H_2_edta, pH 5.6 [24,30]. A summary of *λ*_max_, *ε*_max_ (UV-vis) and *K*_D_ (BLI) values is given in Table 2. All the data were collected at 295 K.

The experimental data for the binding of Ru(III)–Tf and Ru(III)–Fe(III)–Tf adducts (samples A1–A9 in Table 1) to recombinant human TfR1 from BLI [24,27,30,31], UV-vis spectroscopy and particle size analysis are summarized in Table 2 and Figure 1.

**Table 2:** Summary of UV-Vis, BLI and mean particle size data for Fe(III)–Tf and Ru(III)–Tf adducts ^a^.

Sample ^b^	*λ*_max_, nm	*ε*_max_, M^−1^ cm^−1^	*K*_D_, nM ^c^	Mean Particle Size, nm
apoTf	280	8.2 × 10^4 d^	43	7 ± 1
Ru_2_Tf (A1)	380 ^e^	7.0 × 10^2^	162	7 ± 1
Fe_2_Tf (A2)	470	4.9 × 10^2 d^	4.6 ^f^	7 ± 2
monoFeTf (A3)	460	2.5 × 10^2^	19	7 ± 1
RuFeTf (A4)	460	2.8 × 10^2^	31	6.5 ± 1
monoFeTf + 1.0 eq. KP1019 (A5)	290	1.4 × 10^4^	no binding	175, 805
apoTf + 2.0 eq. KP1019 (A6)	290	2.0 × 10^4^	no binding	450, 970
apoTf + 4.0 eq. KP1019 (A7)	290	2.4 × 10^4^	no binding	>1000
Fe_2_Tf + 1.0 eq. KP1019 (A8)	460	6.2 × 10^2^	5	7 ± 2
Fe_2_Tf + 5.0 eq. KP1019 (A9)	620	7.5 × 10^2^	10	9 ± 2

^a^ See Figure 1 and Figure S1 for details. ^b^ Numbers in parentheses correspond to Table 1. ^c^ Dissociation constants (*K*_D_) for the binding of metal–Tf complexes to TfR1 were calculated using the 1:1 binding model in BLItz software [24,30]. Lower *K*_D_ values correspond to stronger binding. ^d^ These values agreed with those reported in the literature [41]. ^e^ This value is consistent with that reported in the literature (broad absorbance band at 300–400 nm) for Ru_2_Tf [40]. ^f^ This value agrees with that reported in the literature for the Fe_2_Tf–TfR1 binding constant (*K*_D_~5 nM for the 1:1 binding model) [44,45].

The UV-vis spectra of apoTf, Fe_2_Tf and Ru_2_Tf (Figure 1a–c) were consistent with those reported in the literature [40,41]. The BLI data for Fe_2_Tf (black lines in Figure 1d–f) represent a functional model of the Tf cycle in a cell-free system [24,27]. This includes the following: (i) strong binding of Fe_2_Tf to TfR1, which is immobilized on a BLI probe that models the cell surface (step A, pH 7.4); (ii) slow dissociation of Fe_2_Tf from TfR1 under extracellular conditions (step B, pH 7.4); (iii) removal of Fe(III) from Fe_2_Tf under the endosome-mimicking conditions (step C, pH 5.6); and (iv) rapid dissociation of the formed apoTf from TfR1 when the endosome is returned to the cell surface (step D, pH 7.4) [24,27]. The dissociation constant for the Fe_2_Tf–TfR1 system (*K*_D_ = 4.6 nM using a 1:1 binding model, Table 2) was consistent with the literature data [44,45] and with our previous results [24,27,30,31]. Detailed kinetic analysis of BLI data [27,31] was out of scope of this work.

As reported previously [24,27,44,45], binding of apoTf to TfR1 was weak compared with that of Fe_2_Tf (blue line in Figure 1d). The measured value of *K*_D_ was 43 nM (Table 2), although this value likely reflected the partial Fe-saturation of Tf by the trace amounts of Fe present in the buffer [27]. Notably, the binding of Ru_2_Tf to TfR1 was even weaker than that of apoTf (red line in Figure 1d; *K*_D_ = 162 nM in Table 2). This result is consistent with the earlier observation [24] that Ru(III) binding to the Fe(III)–binding sites of Tf disrupts the Tf binding to TfR1. Mean particle sizes in apoTf, Fe_2_Tf or Ru_2_Tf solutions (10 μM Tf in the binding buffer) were ~7 nm according to DLS measurements (Table 2 and Figure S1 in Supplementary Material), which indicates the existence of monomeric protein units in solution [46]. Taken together, these data confirm the early report [40] that the reaction of apoTf with [Ru(nta)_2_]^3−^ in dilute aqueous solutions (A1 in Table 1) leads to strong and selective Ru(III) binding to the Fe(III) binding sites of apoTf.

The formation of monoFeTf (a mixture of Fe*_C_*Tf and Fe*_N_*Tf forms) [28] by the reaction of apoTf with one molar equivalent of [Fe(nta)_2_]^3−^ (A3 in Table 1) was confirmed by UV-vis spectroscopy [41] and BLI data (blue lines in Figure 1b,e). As expected [27], the binding of monoFeTf to TfR1 (*K*_D_ = 19 nM, Table 2) was weaker than that of Fe_2_Tf (*K*_D_ = 4.6 nM, Table 2). Reaction of pre-formed monoFeTf with one equivalent of [Ru^III^(nta)_2_]^3−^ (A4 in Table 1) led to spectral changes that indicated the formation of mixed Ru(III)–Fe(III)–Tf adducts. This was consistent with further decrease in strength of Tf–TfR1 binding (red lines in Figure 1b,e; *K*_D_ = 31 nM, Table 2), which was closer to that of apoTf (*K*_D_ = 43 nM) than Fe_2_Tf. Formation of mixed Tf adducts with Fe(III) and exogenous metal ions, such as Ru(III), is the most likely scenario in biological systems [31,47]. Both monoFeTf and RuFeTf adducts retained monomeric states in solution (mean particle size ~7 nm, Table 2 and Figure S1). By contrast, the reaction of pre-formed monoFeTf with one molar equivalent of KP1019 (A5 in Table 1 and Table 2) led to a strong decrease in absorbance at ~280 nm and to complete disruption of Tf–TfR1 binding (green lines in Figure 1b,e). These changes corresponded to a drastic increase in mean particle size (two peaks at ~175 nm and ~805 nm, Table 2 and Figure S1), which pointed to protein aggregation [46]. Similarly, the reactions of apoTf with 2–4 molar equivalents of KP1019 (A6 and A7 in Table 1 and Table 2) led to decreases in UV-vis absorbance intensity, complete disruption of Tf–TfR1 binding and a drastic increase in mean particle sizes (Table 2). In addition, reactions of apoTf with 0.5–1 molar equivalents of KP1019 led to smeared patterns in urea gel electrophoresis (Figure S2 in Supplementary Material), which pointed to disruptions to the protein conformation [29]. Partial protein aggregation during the reactions of apoTf with KP1019 was reported previously [32], but the effect was weaker than that observed in this work, probably because of the differences in reaction conditions.

In contrast with the results for monoFeTf, reaction of Fe_2_Tf with one molar equivalent of KP1019 (A8 in Table 1 and Table 2) did not cause drastic changes in UV-vis spectra, Tf–TfR1 binding patterns or protein particle size (see blue lines in Figure 1c,f and numerical values in Table 2). Urea gel electrophoresis under these conditions showed a slight smear compared with a sample of Fe_2_Tf (Figure S2). Reaction of Fe_2_Tf with five molar equivalents of KP1019 (A9 in Table 1 and Table 2) produced a green-colored solution that showed an additional UV-vis absorbance band at ~630 nm (red line in Figure 1c), a slightly decreased Tf–TfR1 binding affinity compared with Fe_2_Tf (red line in Figure 1f; *K*_D_ = 10 nM, Table 2) and a slightly increased mean particle size in DLS measurements (~9 nm, Table 2 and Figure S1). The green color was similar to that observed previously in the decomposition products of KP1019 in the presence of proteins, although the structures of these species have not been elucidated [36]. These features are consistent with non-specific Ru(III) binding to surface His residues of Fe_2_Tf [25,26], which does not lead to major disruptions within the Tf cycle [27].

Importantly, all the changes shown in Figure 1 and Table 2 were observed during the reactions of Tf with Ru(III) complexes in an aqueous buffer (20 mM HEPES, 25 mM NaHCO_3_, 140 mM NaCl, pH 7.4) [27] that did not contain added proteins. Mixing of apoTf (30 μM) with KP1019 (60 μM) in cell culture medium (DMEM supplemented with ~15 μM BSA) did not have a major effect on Tf–TfR1 binding compared with the same medium that contained added apoTf but not KP1019 (Figure S3a in Supplementary Material). In both cases, BLI curves corresponded to those of partially Fe(III)-saturated Tf due to significant background Fe levels in the medium (~6 μM as measured by GFAAS). These results were in stark contrast with those observed in aqueous buffers, where the addition of KP1019 to apoTf or monoFeTf completely prevented Tf–TfR1 binding (Table 2 and Figure 1e). Furthermore, incubation of whole human serum that naturally contains ~30 μM Tf and 0.60 mM HSA [42] with 100 μM of typical RuACDs (NAMI-A, KP1019 or RAPTA-C) [1,4,16] did not have any effect on Tf–TfR1 binding, while the addition of Fe(III) (60 μM, as citrate) to serum significantly increased the binding affinity (Figure S3b), in agreement with previously published data [27]. These results show that KP1019 is more likely to react with other components of serum or cell culture medium, such as albumin or amino acids [11,48], compared with Tf. By contrast, Fe(III) ions are able to bind selectively to the vacant Tf binding sites under these conditions.

### 3.2. Effect of Tf on the Uptake of Ru Complexes by HepG2 Cells

To further investigate the role of Tf in the uptake of RuACDs, human liver cancer (HepG2) cells were used to study cellular uptake of KP1019, [Ru(nta)_2_]^3−^ (a reference Ru(III) complex) or isolated Ru_2_Tf. Cellular Fe levels were also measured to control the Tf-mediated Fe uptake [30]. The experiments were designed based on similar protocols as those published for Cr(III) uptake by HepG2 cells in the presence of Tf [30], and the results are summarized in Table 3. Most of the conditions (B2-B9 in Table 1 and Table 3) were relatively non-toxic, as judged from the absence of significant changes in cell morphology. Note that in these cases KP1019 was allowed to decompose and bind to the components of cell culture medium for 24 h prior to the cell treatment, which led to reductions in cellular Ru uptake and cytotoxicity [36]. One exception was the condition B1 (KP1019 freshly added to cell culture medium), which led to significant cell detachment during the incubation due to the toxicity of KP1019.

In agreement with previous data [36], treatment of cells with freshly prepared KP1019 solution in cell culture medium (60 μM Ru for 24 h) resulted in significant Ru uptake (B1 in Table 3), while decomposition of KP1019 in cell culture medium for 24 h prior to the addition to cells resulted in a ~30-fold decrease in Ru uptake (B2 in Table 3). The addition of HSA (30 μM) resulted in a further decrease in Ru uptake compared with the basal medium (B3 in Table 3), due to Ru binding to HSA [48] that reduced its bioavailability to cells. This finding parallels the corresponding findings for the Cr(III) uptake experiments, whereby the interaction between HSA and Cr(III) resulted in a decrease in Cr(III) uptake [30]. Condition B3 resulted in the lowest cellular uptake of Ru of all the studied experimental conditions, except for untreated cells where no detectable Ru levels were found (Table 3).

Measurements of Ru–protein binding in cell culture medium after the cell treatment (Table 3) showed that ~50% of KP1019 decomposition products were protein-bound even in the basal medium that contained ~15 μM BSA (conditions B1 and B2 in Table 3). The addition of a minimal amount of BSA to the medium was required to protect cell viability during the treatments [30]. The presence of added HSA, apoTf, or Fe_0.6_Tf, but not Fe_2_Tf (30 μM protein in all cases; conditions B3-B6 in Table 3) resulted in further increases in Ru–Tf binding, while the highest level of Ru–protein binding was observed for the isolated Ru_2_Tf (condition B9 in Table 3). These results are consistent with preferential Ru(III) binding to albumin, or to vacant Fe(III)-binding sites of Tf, rather than to the side chains of Fe(III)-saturated Tf [49,50,51].

**Table 3:** Ru and Fe uptake by HepG2 cells and Ru–protein binding in cell culture medium in the presence or absence of Tf (24 h assays) ^a^.

Conditions ^b^	Ru Uptake ^c^	Fe Content ^c^	% Ru–Protein ^d^
no Ru added	0	1.9 ± 0.7	0
KP1019 fresh (B1)	6.6 ± 2.8 ^e^	11.2 ± 6.5 ^f^	46 ± 4
KP1019 decomposed (B2)	0.23 ± 0.09 ^e^	1.2 ± 0.8	49 ± 1
KP1019 + HSA (B3)	0.062 ± 0.005	1.6 ± 0.5	73 ± 3 ^g^
KP1019 + apoTf (B4)	0.13 ± 0.04 ^e^	1.3 ± 0.6	64 ± 1 ^g^
KP1019 + Fe_0.6_Tf (B5)	0.13 ± 0.03 ^e^	3.4 ± 3.5	64 ± 1 ^g^
KP1019 + Fe_2_Tf (B6)	0.26 ± 0.03 ^e^	8.9 ± 1.7 ^f^	53 ± 4
[Ru(nta)_2_]^3−^ (B7)	0.09 ± 0.03	1.4 ± 0.4	2 ± 1 ^g^
[Ru(nta)_2_]^3−^ + Fe_2_Tf (B8)	2.9 ± 0.3 ^e^	5.8 ± 1.7 ^f^	3 ± 1 ^g^
Ru_2_Tf (B9)	0.07 ± 0.06	1.9 ± 0.7	74 ± 3 ^g^

^a^ Mean values and standard deviations of three replicate measurements; see Figure 2 for graphical representation. ^b^ Numbers in parentheses correspond to Table 1. The basal medium used was serum-free DMEM, supplemented with 1.0 mg mL^−1^ (~15 μM) BSA; concentrations of added HSA or Tf were 30 μM, and that of added Ru was 60 μM [30]. ^c^ Cellular metal content was measured in nmol per mg protein. ^d^ Proportion of protein-bound Ru in cell culture medium (molar %), measured by gel-filtration chromatography [30,31]. ^e^ Statistically significant difference (*p* < 0.001 for B2 and B8; *p* < 0.01 for B4–B6) compared with condition B3. ^f^ Statistically significant difference (*p* < 0.05) compared with untreated cells. ^g^ Statistically significant difference (*p* < 0.001 for B7 and B8; *p* < 0.05 for B3–B5 and B9) compared with condition B2.

Decomposition of KP1019 in cell culture medium in the presence of apoTf, partially Fe(III) saturated Tf (Fe_0.6_Tf), or Fe_2_Tf (condition B4–B6 in Table 3), caused a significant (*p* < 0.01) increase in Ru uptake, compared with KP1019 decomposed in the presence of HSA (condition B3). However, in all these cases, the Ru uptake did not exceed the Ru uptake level when KP1019 was decomposed in the absence of added Tf (condition B2). Furthermore, Ru uptake from isolated Ru_2_Tf was very low (condition B9 in Table 3) and comparable with that of KP1019 decomposed in the presence of HSA (condition B3). These data showed that Ru_2_Tf did not play an active part in Ru transport into cells, which is similar to the previous findings for Cr_2_Tf and cellular Cr uptake [30].

In agreement with the previous data [30], the presence of Fe_2_Tf (conditions B6 and B8 in Table 3), but not of apoTf or Fe_0.6_Tf (conditions B4 and B5 in Table 3) significantly (*p* < 0.05) increased cellular Fe content over the basal level, due to active Fe_2_Tf transport into the cells via the Tf cycle [27]. Cellular Fe levels were also increased for experiments conducted under condition B1 (Table 3), possibly due to the protective reaction of cells to KP1019 toxicity [52]. Most notably, co-treatment of HepG2 cells with [Ru(nta)_2_]^3−^ (60 μM) and Fe_2_Tf (30 μM) resulted in a highly significant (*p* < 0.001) increase in cellular Ru levels at a very low level of Ru–protein binding (condition B8 in Table 3). This finding parallels the previous observation [30] of high cellular Cr(III) (CrCl_3_·6H_2_O equilibrated with cell culture medium) uptake in the presence of added Fe_2_Tf, which did not result in significant Cr(III)–Tf binding. Contrary to previous suggestions [25,26], these results indicate that covalent Ru binding to the side chains of Fe_2_Tf is not required for Tf-mediated cellular Ru uptake (discussed in Section 4.4).

**Figure 2:**
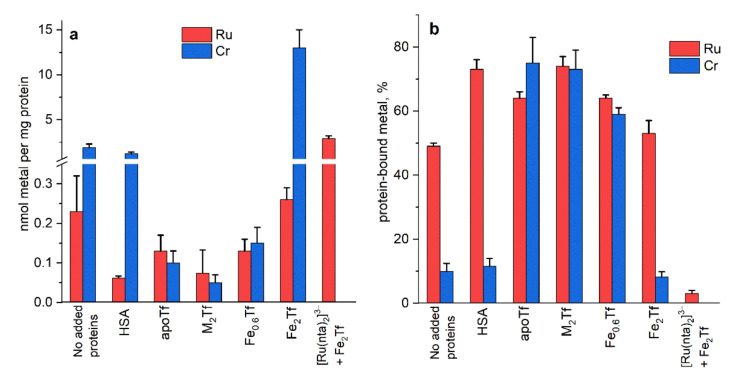
Comparison of cellular Ru uptake (**a**) and proportions of protein-bound Ru(III) (**b**) from Table 3 with the corresponding values for Cr(III) [30]. Unless indicated otherwise, the source of Ru(III) was KP1019 and that of Cr(III) was CrCl_3_·6H_2_O. In all cases, the metal complexes (60 μM) were pre-equilibrated with cell culture medium (DMEM with ~15 μM BSA) in the presence or absence of added proteins (indicated on the X axis, 30 μM) for 24 h at 310 K and 5% CO_2_. Prepared medium was added to HepG2 cells for further 24 h. The error bars represent mean values and standard deviations of three replicate wells.

To summarize the similarities and differences in Ru(III) and Cr(III) uptake by HepG2 cells, Figure 2 shows: (a) a comparison of cellular metal content; and (b) the proportion of total Ru bound to protein in cell culture medium for Ru(III) (Table 3) with the corresponding published data for Cr(III) [30]. A crucial difference between Ru(III) and Cr(III), as observed in these experiments, was their relative affinity for albumin vs. Tf (Figure 2b). Decomposed KP1019 was strongly bound to albumin (30 µM of added HSA and/or 15 µM of background BSA), while the addition of apoTf or partially Fe-saturated Tf only slightly increased Ru–protein binding (Table 3). By contrast, Cr(III) bound stronger to the vacant Fe(III) binding sites of Tf compared with albumin (Figure 2b). This difference is consistent with the nature of Fe(III) and Cr(III) as hard Lewis acids, and Ru(III) as a softer Lewis acid [53]. Correspondingly, cellular uptake of Ru(III) was strongly suppressed by added HSA (a strong Ru(III) binder) and slightly suppressed by apoTf, or by partially Fe-saturated Tf (weaker Ru(III) binders), while the reverse pattern was observed for Cr(III) (Figure 2a). For both Ru(III) and Cr(III), pre-formed adducts with apoTf (designated M_2_Tf in Figure 2) showed kinetic and thermodynamic stabilities in biological media, as judged from high levels of metal–protein binding in cell culture medium, but were not significantly taken up by cells.

## 4. Discussion

### 4.1. Changing Paradigm in Anticancer Ru(III) Complexes

The initial design and development of KP1019 and related anticancer Ru(III) complexes was based on the “activation by reduction” hypothesis [54,55]. This hypothesis assumed that Ru(III) mirrors cellular uptake pathways of Fe(III) [21], including selective binding to the Fe(III) sites of Tf, efficient transport of Ru(III)–Tf into cells via the TfR1-mediated pathway, and intracellular reduction of Ru(III) to Ru(II) [54,55]. This pathway was expected to lead to enhanced Ru(III) uptake by cancer cells that overexpress TfR1 [56] and selective activation of the drug in the hypoxic environment of solid tumors [57] that facilitate Ru(III) reduction to Ru(II) [54,55]. This hypothesis was subsequently challenged by several experimental findings. First, human pharmacokinetic studies have shown that KP1019 decomposition products in blood serum are bound predominantly to albumin, while Tf binding does not exceed 1–2% of total Ru [54,58]. Second, capillary electrophoresis and X-ray absorption spectroscopic studies have shown that protein-bound Ru(III) is unlikely to convert to Ru(II) under biologically relevant conditions [36,59,60,61]. Finally, there is mounting evidence that binding of non-Fe metals to the Fe(III)-binding sites of Tf disrupts the Tf–TfR1 binding and is likely to lead to exclusion of exogenous metal ions from cells [24,25,30,31,47,62].

These observations, together with recent setbacks in human clinical trials of NAMI-A and KP1019 [1,16], clearly show that better understanding of reactivity of anticancer Ru(III) complexes in biological media is required to achieve further progress in this area. Although the reactions of KP1019 with Tf and other serum proteins have been extensively studied previously (reviewed in refs. [13,51]), application of the BLI technique [24,27] in this study provided novel insights into the roles of Tf in biological activities of KP1019 and other metal-based anticancer drugs.

### 4.2. Interactions of Ru(III) Complexes with Tf in Aqueous Buffers

**Scheme 1:**
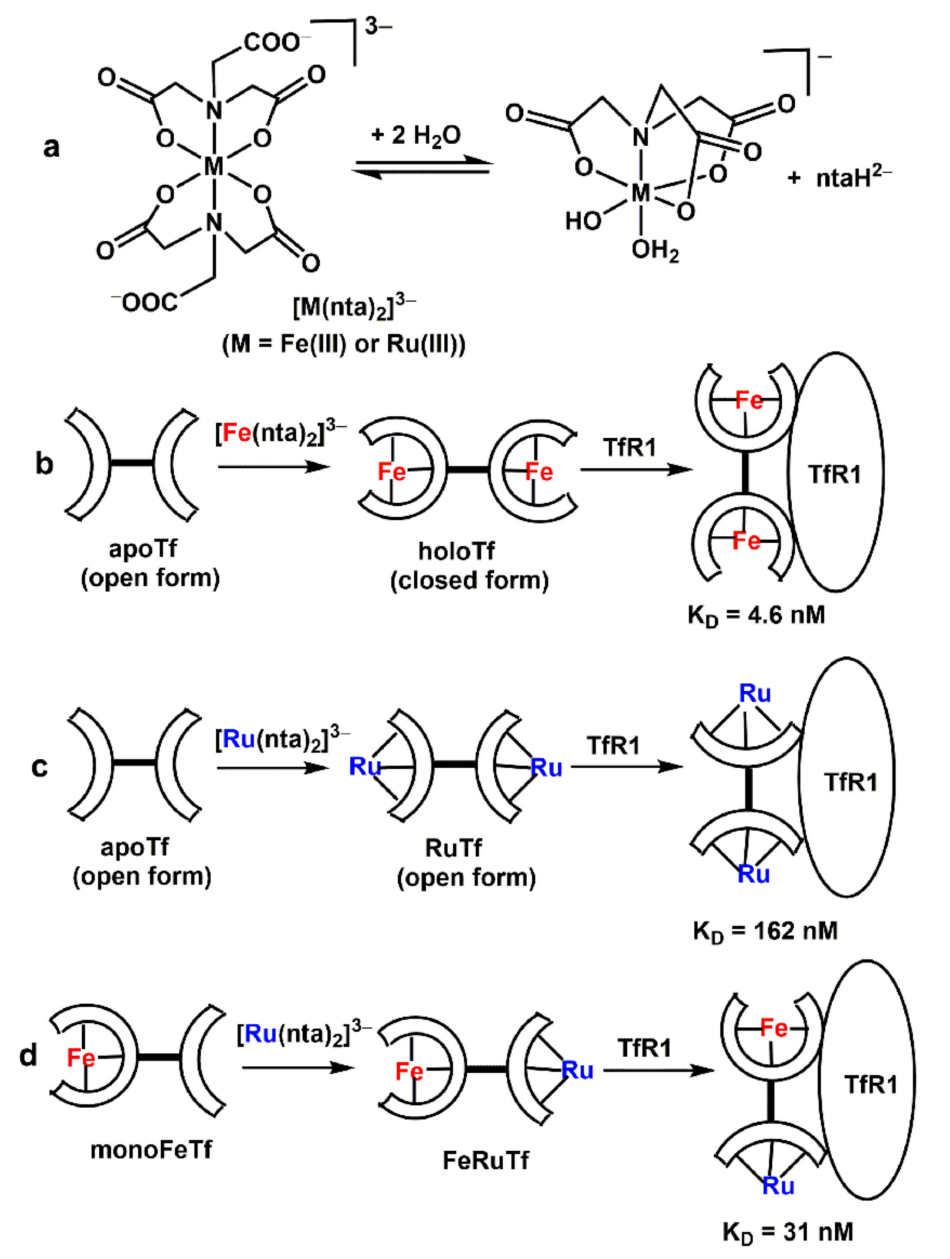
(**a**) Likely equilibrium processes involving Fe(III)– and Ru(III)–nta complexes [39] in neutral aqueous solutions (the structures shown represent one of potential mixtures of geometric isomers). (**b**) Binding of two equivalents of [Fe^III^(nta)_2_]^3−^ to apoTf results in formation of Fe_2_Tf (closed conformation of the protein) that binds tightly to TfR1 [27,44,45]. (**c**) Binding of two equivalents of [Ru^III^(nta)_2_]^3−^ to apoTf [40], which retains an open conformation of the protein and leads to weak Ru_2_Tf–TfR1 binding [24]. (**d**) Binding of one equivalent of [Ru^III^(nta)_2_]^3−^ to monoFeTf, which leads to formation of a mixed-metal Tf complex that partially binds to TfR1 [44,45]. The two lobes of Tf (*C*- and *N*-lobes) are not distinguished in this schematic representation. Values of dissociation constants (*K*_D_, determined by BLI analysis) were taken from Table 2.

Initial in vitro experiments (Table 2 and Figure 1) were performed in aqueous buffers under the conditions that facilitate metal–Tf binding [27,39,40,41] in the absence of blood serum proteins (such as BSA). These studies showed a striking dependence of the mode of Ru–Tf binding on the type of Ru(III) complex and the form of Tf used. In agreement with literature data [40], the use of [Ru(nta)_2_]^3−^ (Scheme 1a), an analogue of a Fe(III) complex that is widely used for apoTf loading with Fe(III) [39], led to Ru(III) binding to the Fe(III) binding sites of apoTf, as shown by UV-vis spectroscopy (Figure 1a).

Binding of two equivalents of Fe(III) to apoTf is well known to lead to Tf folding into a closed conformation that promotes tight association to TfR1 (Scheme 1b) [27,28,44,45]. No such folding occurs in the case of Ru(III)–Tf adducts [24,40], which led to weak Ru(III)–Tf binding to TfR1 (Scheme 1c), as was confirmed by BLI data (Table 2 and Figure 1d). In addition, binding of one equivalent of Ru(III) to partially Fe(III)-loaded Tf did not increase the affinity of Tf–TfR1 binding (Scheme 1d and Table 3), contrary to suggestions in the literature [31,47,63] that such mixed-metal Tf complexes may be particularly favorable for cellular uptake. Note that the binding of Ru(III) from [Ru(nta)_2_]^3−^ to the Fe(III) binding sites of Tf (Scheme 1c,d) [40] is inconsistent with recent X-ray crystallography data [26] that showed non-specific binding of Ru(III)–nta to the surface His residues of Tf. However, this result is likely to be due to the conditions of crystal growth, which included soaking of pre-formed monoFeTf crystals in a Ru(III)-containing buffer [26], where binding to surface histidines was more kinetically favored over the multiple steps and conformational changes required to bind to the Fe(III) site. Using preformed crystals can lead to difference in coordination sites within proteins compared to when crystals are grown from solution, as discussed previously [64]. Examples of binding of non-Fe metal ions to the Fe(III) binding sites of Tf that retained open or partially open Tf conformation (confirmed by X-ray crystallography) include Bi(III) [65] and Ti(IV) [66].

**Scheme 2:**
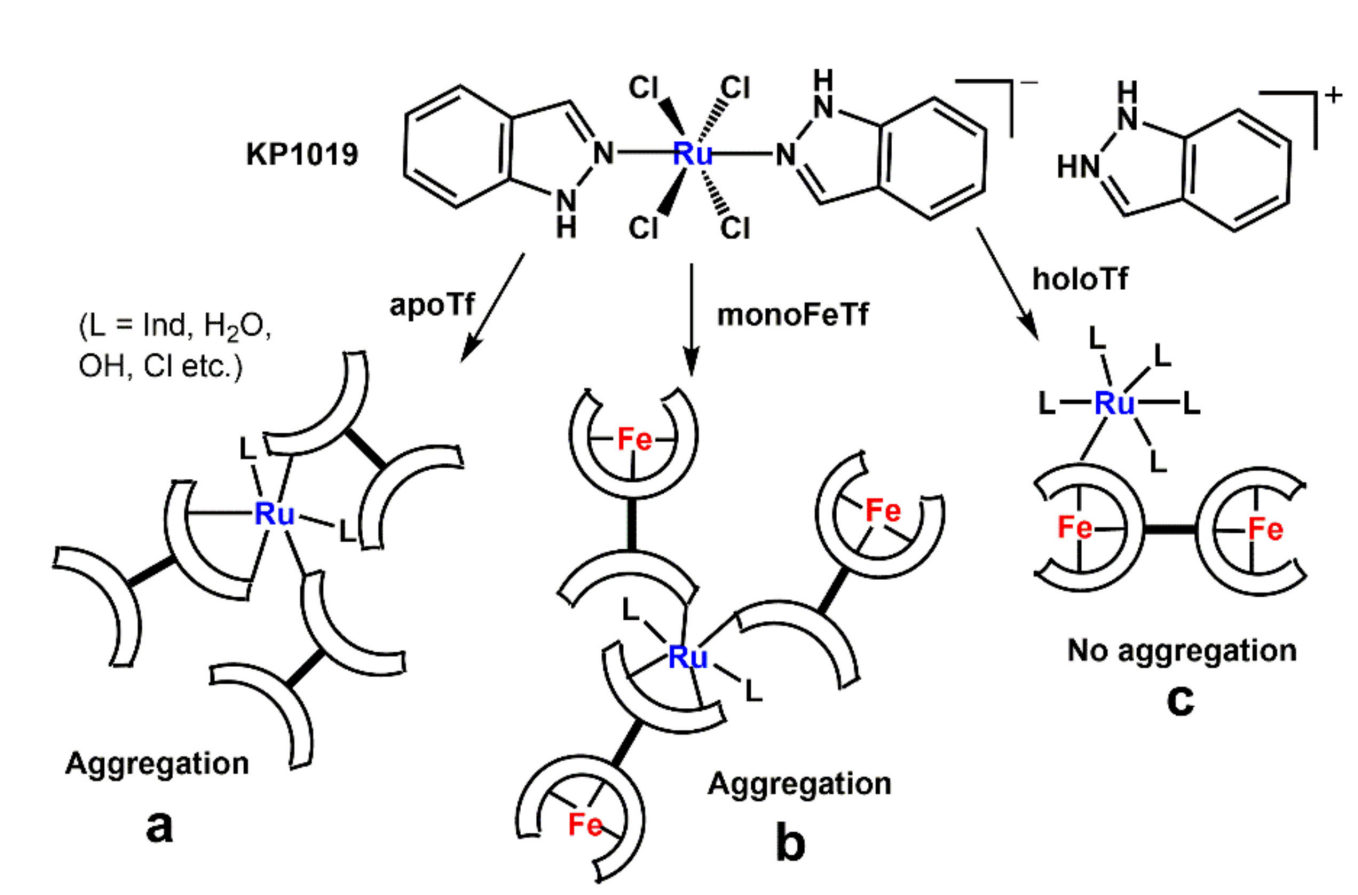
Proposed reaction modes of KP1019 with open (**a**), partially open (**b**) or closed (**c**) forms of Tf in aqueous buffers (pH = 7.4) in the absence of added albumin.

Reactions of KP1019 with apoTf or with monoFeTf in an aqueous buffer in the absence of added proteins led to extensive protein aggregation and loss of activity (Table 1 and Figure 1b,e), as shown in Scheme 2. This aggregation was probably caused by ligand-exchange reactions of Cl^−^ ligands of KP1019 with a range of potential surface binding sites in Tf molecules, which would have led to protein cross-linking (Scheme 2a,b) [10,11,13,36]. On the other hand, reactions of one to five equivalents of KP1019 with Fe_2_Tf, which resulted in Ru–Tf binding (evident from UV-Vis spectroscopic data, Figure 1c), did not lead to significant protein aggregation (as shown by particle size analysis, Table 2), and had a relatively minor effect on Tf–TfR1 binding (Table 2 and Figure 1f). These observations suggested that Ru(III) from KP1019 can bind to the surface L-histidine (His) residues of Fe_2_Tf (Scheme 2c), as was found in X-ray crystallographic analysis of KP1019 adducts with human serum albumin [48].

**Figure 3:**
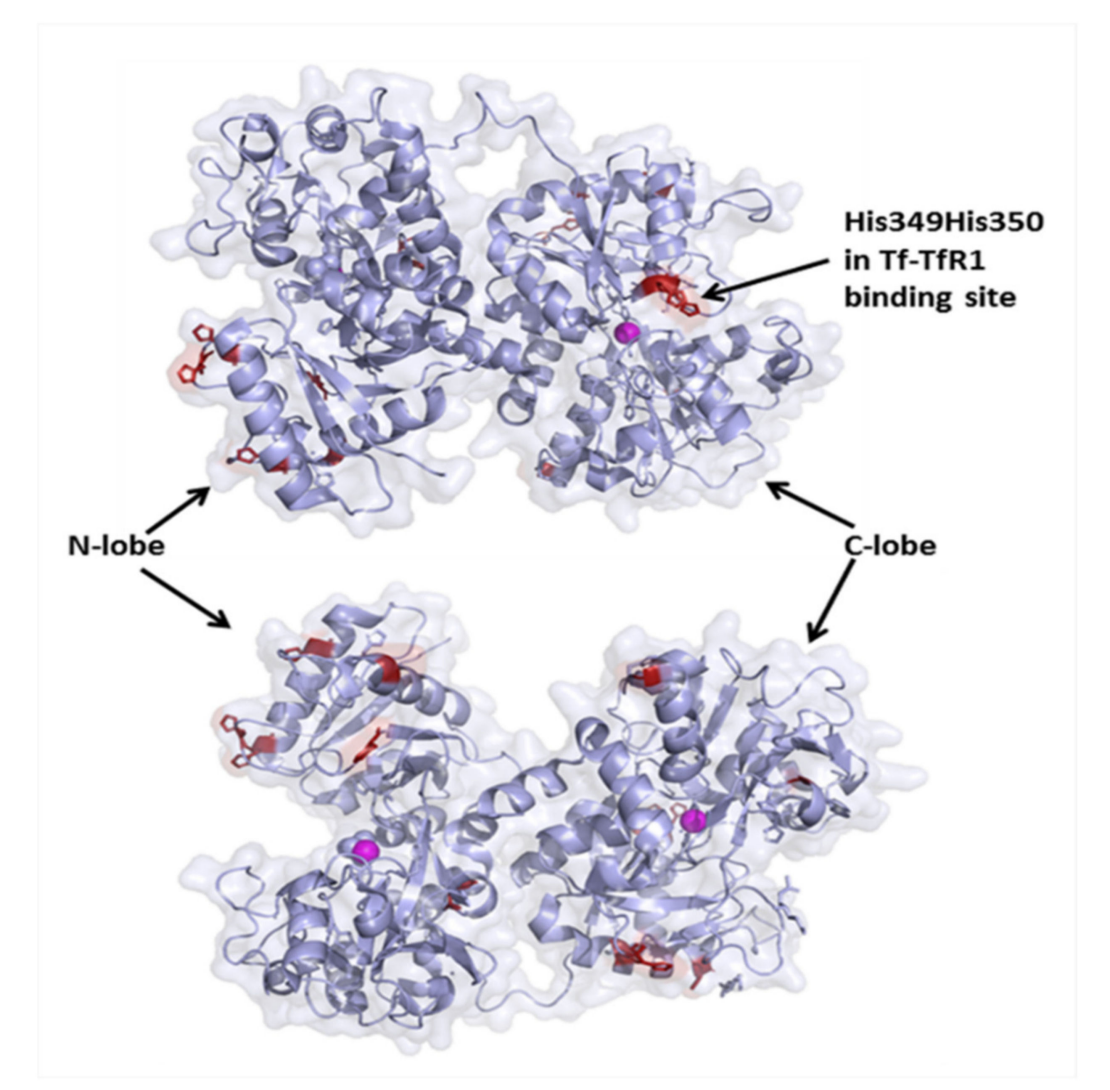
Two views (front and back) of the crystal structure of holoTf (PDB entry 3QYT, processed with PyMOL software) [65]. Pink spheres are the Fe(III) ions, and His residues that lie close to the protein surface and are likely to be available for Ru(III) binding [26,67] are shown in red. The marked His residues (349 and 350) occur at the C-lobe binding site to TfR1 [69].

Our re-analysis of a published crystal structure of human Fe_2_Tf (PDB entry 3QYT; Figure 3) [65] revealed at least thirteen likely surface His residues (marked with red color in Figure 3), in agreement with published data [67]. Notably, one of these sites that contains two adjacent His residues (His349H350, marked in Figure 3) plays a crucial role in the binding of *C*-lobe of holoTf to TfR1, and primes it for the release of Fe(III) under endosomal condition (through the protonation of His at pH = 5.6) [68,69]. Since the His349His350 site is only moderately accessible for Ru(III) binding [26], the reaction of Fe_2_Tf with one equivalent of KP1019 did not result in significant disruption to the Tf–TfR binding (Table 2 and Figure 1f). However, as the number of KP1019 equivalents increased, the probability of binding to this site increased. Such binding to this crucial site as the KP1019-Fe_2_Tf ratio increased would have led to disruption of Fe_2_Tf–TfR1 binding. This could explain the decreased binding affinity to TfR1 of Fe_2_Tf after treatment with five vs. one equivalent of KP1019 (Table 2 and Figure 1f). Scheme 2 illustrates the likely reason for the observed difference in KP1019 binding to apo- and holo-forms of Tf, as open protein conformation and the availability of additional His residues in Fe(III) binding sites of apoTf [26] favors protein crosslinking.

### 4.3. Interactions of Ru(III) Complexes with Tf in Cell Culture Medium and in Blood Serum

One of the advantages of BLI studies of Tf interactions with metal-based drugs is the ability to conduct experiments in biologically relevant fluids, such as cell culture medium, or intact human serum, to probe the effects of metal complexes on Tf–TfR1 binding [27,30,31]. In this work, as well as in the previous studies [30,31], cell culture medium that was collected after the metal uptake experiments (Table 3) was used directly for BLI studies (after the dilution to [Tf] ~1 µM). The results (Figure S2a in Supplementary Material) showed that a small amount of background protein in the medium (~15 μM BSA; 0.5 molar equivalent relative to Tf) [30] largely restored the Tf–TfR1 binding in the presence of 30 μM apoTf and 60 μM KP1019, which was completely suppressed in the absence of BSA (Table 2 and Figure 1e). These results show that albumin has a higher capacity to bind KP1019 compared with Tf, even when the former protein is present at a lower molar ratio. This provided strong evidence that Ru(III)–Tf binding is unlikely to be responsible for cellular metal uptake under typical cell culture conditions of up to 10% vol. bovine serum added to the medium (up to 60 μM albumin) [11], or indeed from the blood stream from intravenous administration. Measurements of cellular Ru uptake from the media that contain added Tf, but not albumin [26,63], are physiologically irrelevant and can result in experimental data that are not relevant to the clinical setting.

Typical human serum contains ~0.6 mM albumin and ~0.1 mM immunoglobulins, as well as hundreds of less abundant proteins [70], including ~30 µM Tf, of which ~1–10 µM are in the holoTf form and ~10–20 µM are in the monoFeTf form (the values vary dependent on the nutritional Fe status) [42]. Typical peak Ru concentrations in the blood of patients treated with KP1019 in clinical trials were 0.1–0.4 mM [71]. Each HSA molecule contains at least two surface His residues that readily bind KP1019 [48]. These data explain why albumin easily outcompetes Tf for the binding of KP1019, or of two other common RuACDs (NAMI-A and RAPTA-C) [33,35] in serum. This was shown experimentally by the absence of significant changes in Tf–TfR1 binding curves for dilute human serum samples, which were pre-treated with Ru complexes under clinically relevant conditions (0.10 mM for 4 h at 310 K) [71] before dilution (Figure S2b in Supplementary Material).

In summary, comparison of the results of BLI studies of Tf–TfR1 binding in cell culture medium or in dilute human serum (Figure S2) with those for aqueous buffers (Figure 1) provided a good illustration of how important the components of biological media, particularly proteins, can be in affecting the interactions of metal-based drugs with their biological targets [10,11].

### 4.4. Roles of Tf in Cellular Ru Uptake

**Scheme 3:**
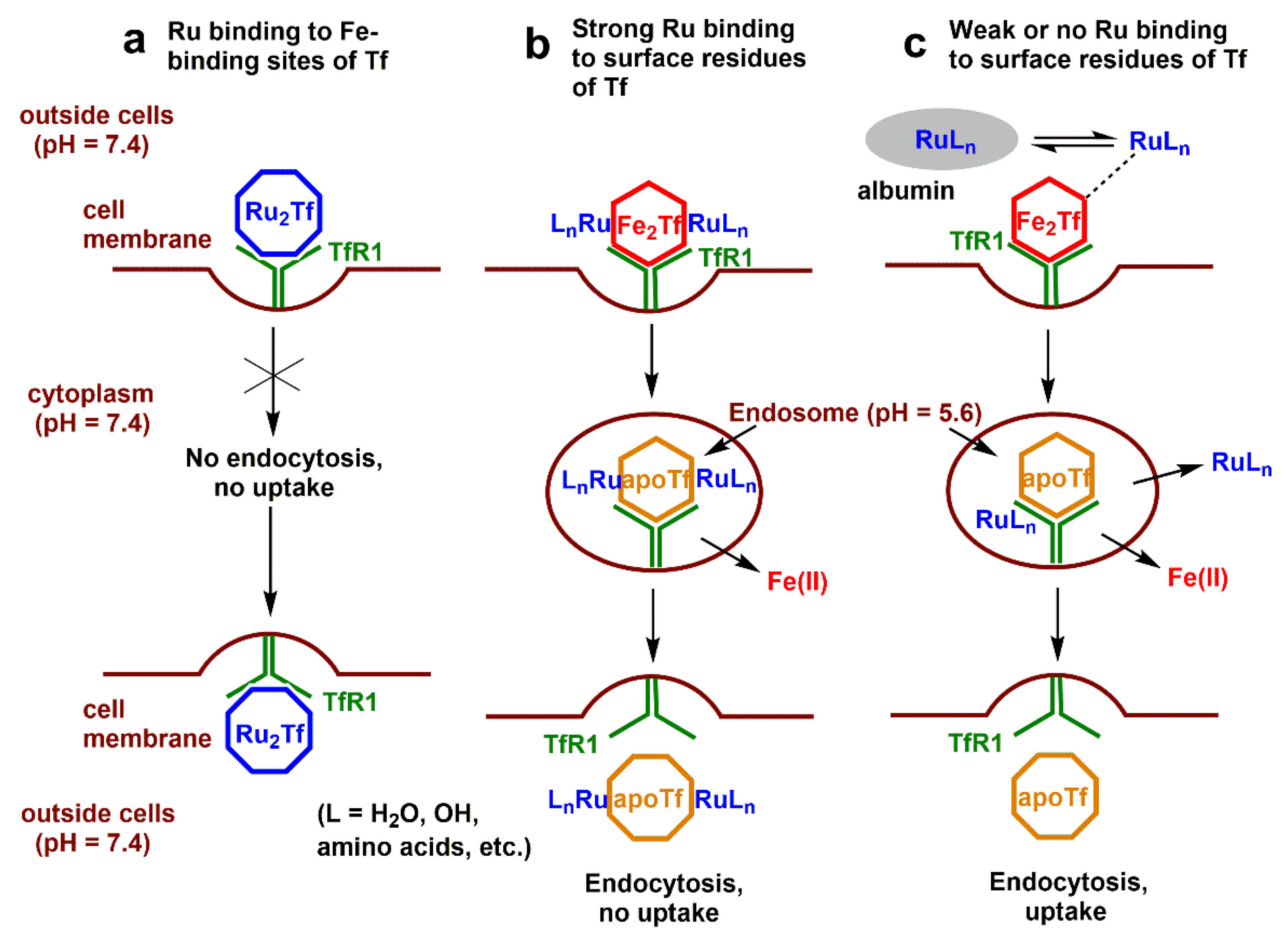
Potential mechanisms that can lead to Tf-mediated cellular Ru uptake: (**a**) selective Ru(III) binding to Fe(III)-binding sites of Tf; (**b**) strong covalent binding of Ru complexes to the surface residues of Tf; and (**c**) trapping of non-covalently bound Ru complexes in the endosome during the endocytosis of Fe_2_Tf.

Scheme 3 summarizes the main possible scenarios of Tf involvement in cellular uptake of RuACDs. Binding of Ru(III) to the Fe(III) binding sites of Tf is likely to retain an open Tf conformation that does not bind tightly to TfR1 (Scheme 3a), as was shown previously for NAMI-A [24] and in this work for [Ru^III^(nta)_2_]^3−^ (Table 2 and Figure 1d,e). The current research also confirmed that pre-formed Ru_2_Tf is not taken up by cells to any significant extent (condition B9 in Table 3), probably because of the blocked endocytosis (Scheme 3a).

Covalent binding of Ru(III/II) complexes [25,26], as well as of other medicinal metal complexes [72], to the surface His residues of Fe_2_Tf has been considered as a likely scenario for cellular uptake of non-Fe metal ions (Scheme 3b). The outcome of this process depends on whether the exogenous metal can dissociate from Tf under endosomal conditions and exit from the endosome to the cytosol [25]. Failure of this process can result in return of Tf-bound metal to the cell surface and low cellular metal uptake (Scheme 3b) [25], as was recently suggested for V(V/IV) complexes [31]. In any case, this is an unlikely scenario for KP1019 because of the absence of significant effect of Fe_2_Tf on cellular Ru uptake or Ru–protein binding (condition B6 in Table 3).

The most remarkable result of cellular Ru uptake studies (Table 3 and Figure 2) is the efficient uptake of [Ru^III^(nta)_2_]^3−^ in the presence of Fe_2_Tf and albumin, neither of which bind the Ru(III) complex to any significant extent (condition B8 in Table 3). As reported previously for Cr(III) uptake [30], the most likely scenario in this case is the trapping of Ru(III) species within the endosomes that are formed on the cell surface following the binding of Fe_2_Tf to TfR1 (Scheme 3c). An increase in Fe_2_Tf concentration increases the extent of Tf-TfR1 endocytosis and, hence, the uptake of drugs by this mechanism. There are few reported examples when cellular uptake of small molecule drugs is enhanced via receptor-mediated endocytosis without the need for such drugs to bind to proteins [73,74]. The mechanism shown in Scheme 3c appears realistic for metal complexes with strong chelating ligands and no leaving groups, such as Ru(II) polypyridyl complexes [7]. Whether or not such complexes can leave the endosome and enter the cytosol (Scheme 3c) via pH-independent mechanisms requires further investigation. This mechanism can also explain the observed decrease in cellular Ru uptake of a stable Ru(II) –phosphine–carboxylato complex by breast cancer cells with silenced TfR1 expression [75], since a large decrease in cellular TfR1 would greatly decrease Tf-TfR1 endocytosis and, hence, drug uptake. By contrast, the mechanisms shown in Scheme 3a,b are unlikely to be realized in whole blood, where the majority of Ru species will be bound to albumin rather than Tf [51,54,58].

### 4.5. Potential Application of KP1019 for Intratumoral Injections

As has been reported previously [36] and confirmed in this work (conditions B1 and B2 in Table 3), decomposition of KP1019 in cell culture medium occurred within several hours at 310 K and led to a dramatic decrease in cellular Ru(III) uptake and cytotoxicity. Recently, limited stability of typical transition metal complexes in biological media has been considered as a potential advantage for direct injections into tumors that are widely trialed for the treatment of inoperable cancers [14,76]. These applications rely on the ability of metal complexes with lipophilic organic ligands to enter cells rapidly via passive diffusion and cause high cytotoxicity, while their decomposition products, such as metal–protein complexes, are much less toxic and can have beneficial effects [14,76]. Potential beneficial effects of Ru(III)–protein adducts [14] can include antimetastatic [16,77], immunomodulatory [8,9,19], and antimicrobial [78] activities [14]. The development of these novel applications is particularly attractive for KP1019 and other anticancer metal complexes that have passed extensive animal testing but failed in human clinical trials [1] because of unfavorable pharmacokinetic properties for the traditional intravenous chemotherapy [14]. Development of suitable nanoformulations for controlled Ru release [79] is likely to be the key to success of this approach [14].

## 5. Conclusions

The nature of interactions of anticancer Ru complexes with transferrin (Tf) is determined by the following: (i) the nature of the complex, particularly the presence of leaving groups, such as Cl^−^; (ii) the availability of Fe(III)-binding sites in Tf; and (iii) the presence of competing proteins for Ru binding, particularly albumin.

Binding of Ru(III) to vacant Fe(III) binding sites of Tf leads to disruption of Tf cycle and is unlikely to cause significant Ru uptake by cells.

Non-specific Ru binding to the surface His residues does not significantly disrupt the Tf cycle and can lead to cellular Ru uptake under the condition that Ru species can dissociate from Tf in the endosome and escape to the cytosol. However, this uptake mechanism is unlikely to occur in the presence of competing proteins, such as serum albumin in the blood, that preferentially bind Ru.

Stable Ru complexes that are incapable of covalent binding to Tf or albumin can be taken into cells by being trapped in the endosome during TfR1-mediated endocytosis of Fe_2_Tf. This is the most likely scenario of Tf-mediated uptake of Ru complexes in vivo.

Complexes with lipophilic organic ligands, such as KP1019, are more likely to be taken into cells intact via passive diffusion rather than by Tf-mediated pathways.

The limited lifetime of KP1019 in biological media can potentially be used to advantage in intratumoral injections.

## Figures and Tables

**Table 1:** Conditions of sample preparation.

Designation ^a^	Description ^b^
A1	apoTf (100 µM) + 2.0 eq. [Ru^III^(nta)_2_]^3−^→Ru_2_Tf
A2	apoTf (100 µM) + 2.0 eq. [Fe^III^(nta)_2_]^3−^→Fe_2_Tf
A3	apoTf (100 µM) + 1.0 eq. [Fe^III^(nta)_2_]^3−^→monoFeTf
A4	apoTf (100 µM) + 1.0 eq. [Fe^III^(nta)_2_]^3−^, then 1.0 eq. [Ru^III^(nta)_2_]^3−^→RuFeTf
A5	apoTf (100 µM) + 1.0 eq. [Fe^III^(nta)_2_]^3−^, then 1.0 eq. KP1019
A6	apoTf (100 µM) + 2.0 eq. KP1019
A7	apoTf (100 µM) + 4.0 eq. KP1019
A8	Fe_2_Tf (84 µM) + 1.0 eq. KP1019
A9	Fe_2_Tf (84 µM) + 5.0 eq. KP1019
B1	KP1019 (60 µM), freshly added to the base medium
B2	KP1019 (60 µM)
B3	KP1019 (60 µM) + HSA (30 µM)
B4	KP1019 (60 µM) + apoTf (30 µM)
B5	KP1019 (60 µM) + Fe_0.6_Tf (30 µM)
B6	KP1019 (60 µM) + Fe_2_Tf (30 µM)
B7	[Ru^III^(nta)_2_]^3−^ (60 µM)
B8	[Ru^III^(nta)_2_]^3−^ (60 µM) + Fe_2_Tf (30 µM)
B9	Ru_2_Tf (30 µM)

^a^ Samples A1–A9 were used for UV-vis, BLI, and DLS measurements (Table 2 and Figure 1) and samples B1–B9 were used for cell uptake and protein binding studies (Table 3 and Figure 2). ^b^ Samples A1–A9 were prepared in the binding buffer (20 mM HEPES, 25 mM NaHCO3, 140 mM NaCl, pH 7.4) and incubated for 24 h at 295 K before the experiments. Samples B1–B9 were prepared in cell culture medium (DMEM supplemented with 1.0 mg mL^−1^ of AlbuMax), using freeze-dried protein samples and 20 mM KP1019 solution in DMF. These samples were sterilely filtered and incubated for 24 h at 310 K and 5% CO_2_ (except for sample B1 that was used immediately after the addition of KP1019).

## Data Availability

The original experimental data are available on request from aviva.levina@sydney.edu.au.

## Supplementary Materials

The following supporting information can be downloaded at: https://www.mdpi.com/article/10.3390/biom12091319/s1, Figure S1: measurements of particle size distribution in Ru(III)–Tf systems by DLS; Figure S2: typical results of urea gel electrophoresis of apoTf and Fe_2_Tf in the presence or absence of KP1019; and Figure S3: typical results of BLI measurements using cell culture medium or human serum as sources of Tf.
