# Supplementary material for: Controversial Role of Transferrin in the Transport of Ruthenium Anticancer Drugs
Source: Biomolecules. 2022 Sep 18;12(9):1319. doi: 10.3390/biom12091319 (PMC9496346; doi:10.3390/biom12091319)
Supplement: Supplementary file 1 [file biomolecules-12-01319-s001.zip › biomolecules-1890452-supplementary.pdf]

Controversial Role of Transferrin In The Transport of Ruthenium Anticancer Drugs

Aviva Levina, Anthony R. M. Chetcuti and Peter A. Lay

Sydney Analytical, and the School of Chemistry, The University of Sydney, Sydney NSW 2006, Australia

Supplementary Material

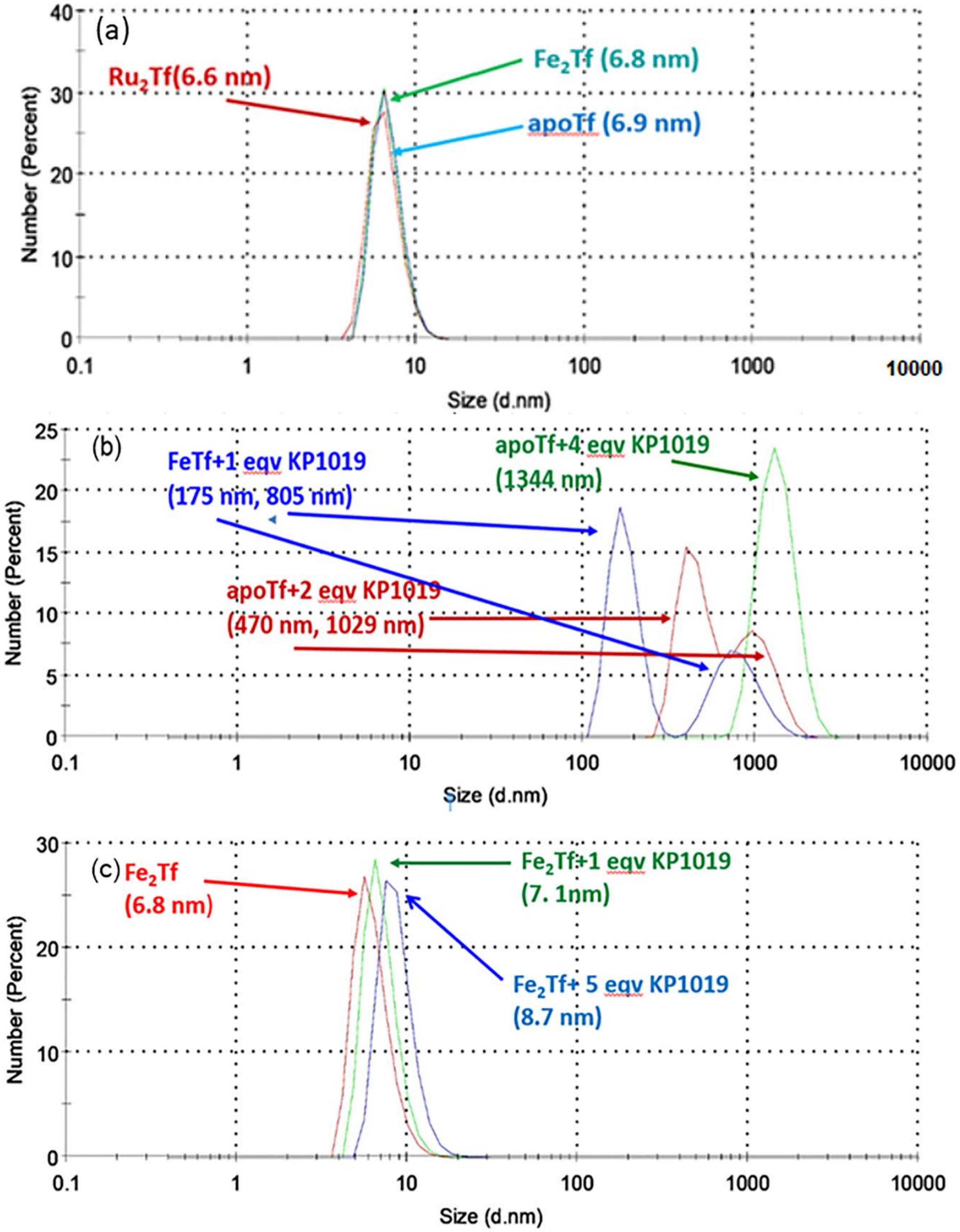

Figure S1. Typical results of particle size distribution measurements by DLS at 298 K. Reaction conditions correspond to those in Tables 1 and 2 (main text): (**a**) apoTf vs. A1 and A2; (**b**) A5, A6, and A7; and (c) Fe_2Tf vs. A8 and A9. The reaction mixtures were diluted 10-fold with the binding buffer (20 mM HEPES, 25 mM $NaHCO_3$, 140 mM NaCl, pH 7.4) immediately before the experiments.

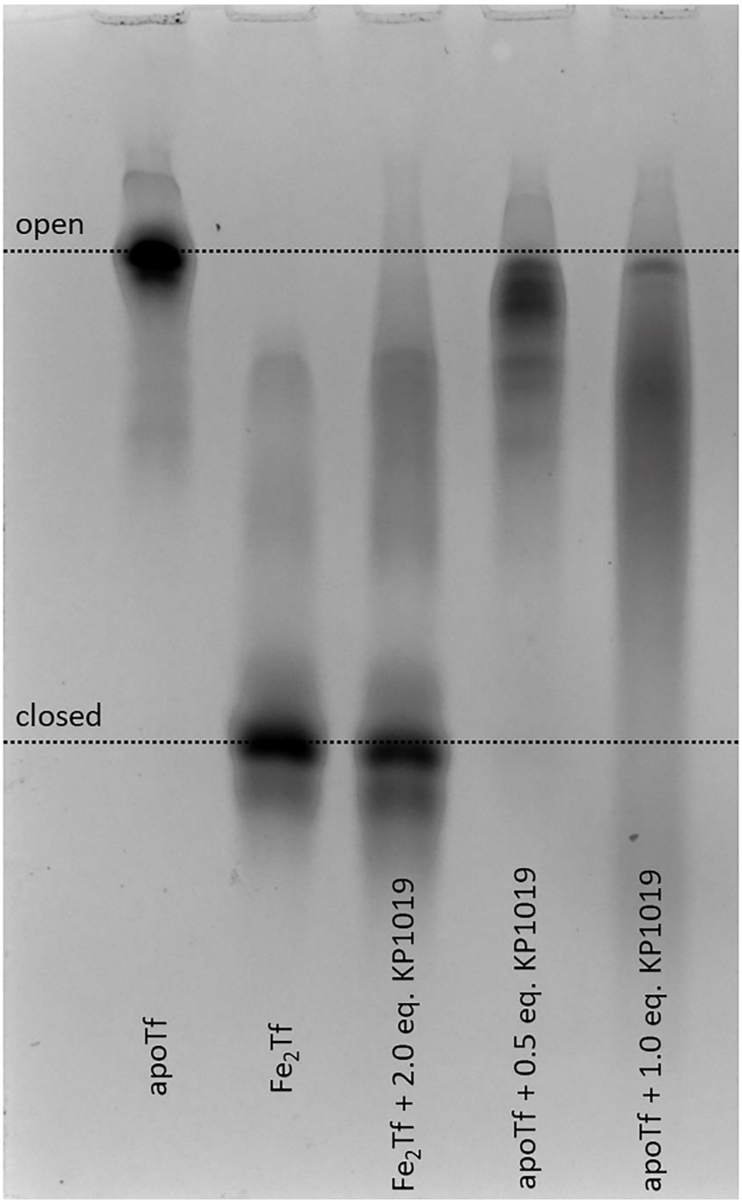

Figure S2. Typical results of urea-PAGE gel electrophoresis of the reaction mixtures containing 30 μM of apoTf or Fe_2Tf in the presence or absence of 15–120 μM of KP1019 in the binding buffer (20 mM HEPES + 25 mM $NaHCO_3$ + 140 mM NaCl, pH = 7.4) after 24 h reaction at 310 K. All samples were diluted 6-fold with the binding buffer before loading into the gel, and gel electrophoresis was performed as described previously [29].

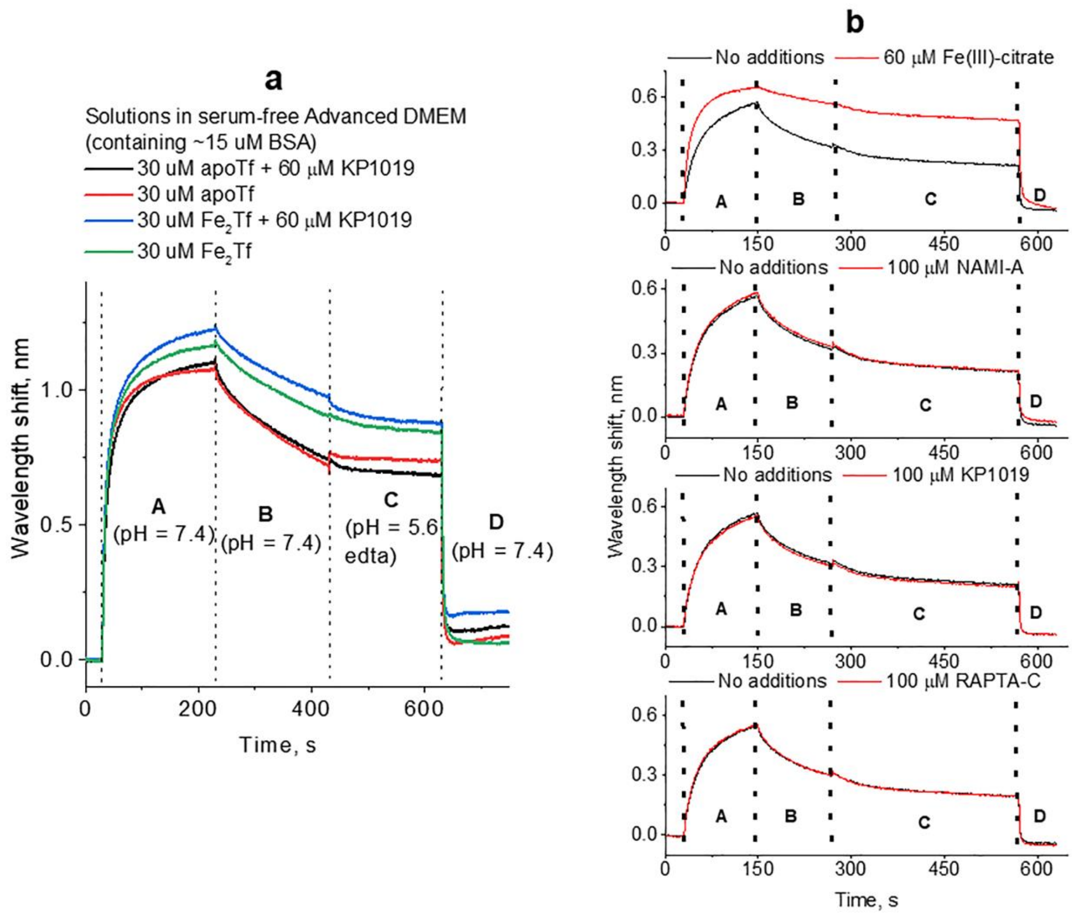

Figure S3.

(a) BLI response curves for 1.0 μM of human Tf at 295 K in dilute cell culture media after Ru uptake experiments by HepG2 cells (Section 3.2 in the main text). Media (serum-free DMEM, containing 1.0 mg mL^{-1} BSA as a background protein, as well as KP1019 and/or Tf, as shown in the figure legend), were collected after the end of uptake experiments, then were diluted 30-fold with buffer (20 mM HEPES + 25 mM $NaHCO_3$ + 140 mM NaCl, pH = 7.4) and applied to TfR1-loaded BLI probe. Designations of the steps in Tf cycle (A–D) correspond to those in Figure 1d-f, main text. Calculated K_D values (1:1 binding model, BLItz software) were as follows: 51 nM (apoTf + KP1019); 32 nM (apoTf); 25 nM (Fe_2Tf + KP1019); 22 nM (Fe_2Tf). Lower Fe_2Tf binding affinity toTfR1 compared with that observed in aqueous buffers (Table 2 in the main text) is likely due to the presence of competing Fe(III) binders, such as phosphate, in cell culture medium [27].

(b) BLI response curves for dilute human serum samples in the presence or absence of metal complexes. Intact commercial human serum (Sigma H4522) was incubated with Fe(III)-citrate (60 μM) or with Ru complexes (100 μM) for 4 h at 310 K, then diluted 200-fold (to [Tf] ~ 150 nM) with the binding buffer (20 mM HEPES, 25 mM $NaHCO_3$, 140 mM NaCl, pH = 7.4), and analyzed with the TfR1-loaded BLI probe. Designations of the steps in Tf cycle (A–D) correspond to those in Figure 1d-f, main text. Calculated K_D values (1:1 binding model, BLItz software) were as follows: 8.0 nM (serum + Fe(III) citrate); or 28 ± 2 nM (serum in the presence or absence of Ru complexes). Typical anticancer Ru complexes [1–6], NAMI-A, KP1019 and RAPTA-C, were synthesized and characterized previously in the Lay group [36,37].
